# Tourette syndrome and other neurodevelopmental disorders: a comprehensive review

**DOI:** 10.1186/s13034-017-0196-x

**Published:** 2017-12-04

**Authors:** Elena Cravedi, Emmanuelle Deniau, Marianna Giannitelli, Jean Xavier, Andreas Hartmann, David Cohen

**Affiliations:** 1 Department of Child and Adolescent Psychiatry, Pitié-Salpêtrière Hospital, APHP, 83, boulevard de l’hôpital, 75013 Paris, France; 2 Pediatric Neurology Unit, Children’s Hospital A. Meyer, University of Firenze, Florence, Italy; 3 Department of Neurology, Reference Centre for Tourette Syndrome, Pitié-Salpêtrière Hospital, APHP, Paris, France; 4 CNRS UMR 7222, Institute for Intelligent Systems and Robotics, Sorbonnes Universités, UPMC, Paris, France

**Keywords:** Tourette, Neurodevelopmental disorder, ADHD, Autism

## Abstract

Gilles de la Tourette syndrome (TS) is a complex developmental neuropsychiatric condition in which motor manifestations are often accompanied by comorbid conditions that impact the patient’s quality of life. In the DSM-5, TS belongs to the “neurodevelopmental disorders” group, together with other neurodevelopmental conditions, frequently co-occurring. In this study, we searched the PubMed database using a combination of keywords associating TS and all neurodevelopmental diagnoses. From 1009 original reports, we identified 36 studies addressing TS and neurodevelopmental comorbidities. The available evidence suggests the following: (1) neurodevelopmental comorbidities in TS are the rule, rather than the exception; (2) attention deficit/hyperactivity disorder (ADHD) is the most frequent; (3) there is a continuum from a simple (TS + ADHD or/and learning disorder) to a more complex phenotype (TS + autism spectrum disorder). We conclude that a prompt diagnosis and a detailed description of TS comorbidities are necessary not only to understand the aetiological basis of neurodevelopmental disorders but also to address specific rehabilitative and therapeutic approaches.

## Background

According to the DSM-5 classification, Gilles de la Tourette syndrome (TS) is a developmental neuropsychiatric disorder characterised by multiple motor and one or more phonic tics, lasting at least 1 year, with onset during childhood or adolescence [1]. A tic is a brief, sudden movement or sound that occurs in an inappropriate context and frequency. The distinguishing characteristics of tic include variability in severity with a waxing and waning course and suppressibility and presence of an anticipatory uncomfortable sensory sensation called a premonitory urge.

Although initially considered to be rare, TS is more common than previously expected, with a suggested overall prevalence of 1/200 in children. TS is reported worldwide in all cultures and is more common in males than females (M/F ratio ranging from 1.6:1 to 9:1) [2, 3]. The aetiopathogenesis of TS is still unclear and more complex than previously perceived, and it involves environmental (infections, perinatal problems, and autoimmunity) and genetic factors that result in a dysfunction of cortico-striato-thalamo-cortical-circuits [4–8].

Comorbidities and coexistent pathologies in TS are also common. Hirschtritt et al. in a large clinical based study, analysed 1374 TS patients and found a lifetime prevalence of any psychiatric comorbidity among 85.7% and that 57.7% of the patients involved at least 2 psychiatric disorders [9]. Moreover, recent factor analysis studies have suggested that TS is not a unitary condition but can be subdivided into more homogeneous components that, similar in phenotype, are suitably share the same genetic background [10, 11]. Because TS comorbidities provide a better understanding of the syndrome not only in terms of classification and aetiopathogenesis but also in terms of outcome, comorbidities are one of the main factors contributing to the psychological and psychosocial impairment observed in TS, often more than the severity of tics [12].

In the DSM-5, TS belongs to the “neurodevelopmental disorders” group, together with intellectual disabilities, communication disorder, autism spectrum disorder, attention deficit hyperactivity disorder, and specific learning disorder. This group includes conditions that co-occur frequently, typically in the early stages of development, and produce deficits in social, personal, academic and occupational functioning [1]. As evidenced by recent literature, TS shares a similar genetic background and risk factors with other neurodevelopmental disorders, that eventually produce similar neuropathological alterations [13, 14]. In particular, recent studies have found similar connectivity alterations among ASD, ADHD and TS patients [6, 15]. Considering all of these findings, the purpose of this article was to provide an extensive review on comorbidity in TS and other neuro-developmental disorders.

## Materials and methods

**Fig. 1:**
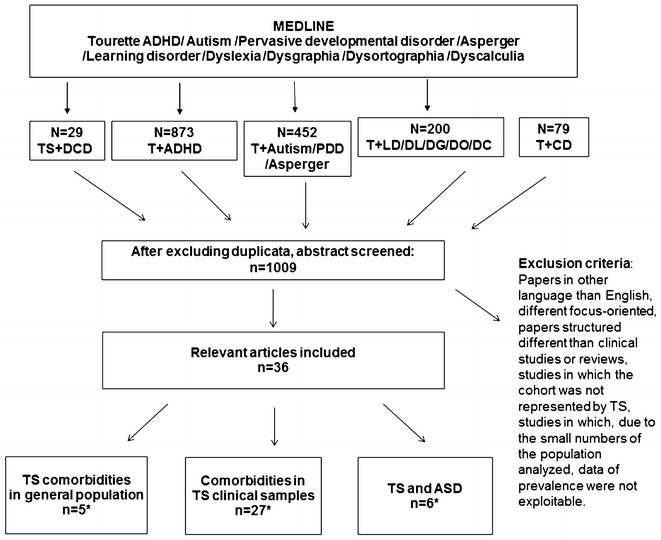
PRISMA diagram flow-chart of the literature search

A Medline (PubMed version) search was performed using the following keywords: Tourette and attention deficit hyperactivity disorder (ADHD) or autism or pervasive developmental disorder or Asperger syndrome or learning disorder or Dyslexia or Dysgraphia or Dysorthographia or Dyscalculia or communication disorders or developmental coordination disorder. We included studies and reviews published in English between 1965 and February 2017. We screened all identified studies or reviews by reading the titles and the abstracts. The inclusion criteria for articles were as follows: the analysis of comorbidity between TS and ADHD/autism (ASD)/learning disorders (LD), in term of prevalence, clinical characteristics and prognosis, resulting from cohorts of TS clinical ascertained samples and population-based studies. Duplicate studies were excluded. To identify any potential study missed by our literature research, we applied, as our second step, a cross-referencing search within retained articles. The initial search sorted out 1633 references. After excluding duplicates, the number was reduced to 1009 reports. Comorbidities between TS and communication disorders or developmental coordination disorder were not analysed due to the lack of detailed literature. In total, 36 papers that fulfilled the study criteria were retained. A PRISMA diagram flow-chart, presented in Fig. 1, summarises the literature selection process and details the primary cause of exclusion. For ease of presentation, we distinguish general population studies (N = 5) and comorbidities in TS clinical-based studies (N = 27) with a specific focus on TS and ASD co-occurrence (N = 6).

## Results

### General population studies

The assessment of comorbidity between TS and other neurodevelopmental disorders has been assessed in several general population-based studies (Table 1). One of the first studies was conducted by Comings and Comings in 1990 [16] in which they evaluated 3034 students in three schools in Los Angeles and reported an approximately 0.46% frequency of TS (N = 14 individuals). Comorbid ADHD was reported in 10 (70%) of 14 students. Apter et al. [17], who screened all recruits in the Israeli Defense Force during a 1-year period, documented 8.3% of ADHD in TS, while the ADHD population point prevalence at that time was 3.9%. The lower prevalence of ADHD in this study is likely due to the age of the subjects (16–17 years old) at the time of evaluation. Wang et al. [18] conducted an epidemiological study in a Taiwanese elementary school with 2000 children, highlighting a 0.56% prevalence of TS and a 36% comorbid rate of ADHD. Kadesjo and Gillberg [19] examined 435 school-age children and found a 0.15% prevalence of TS. Among the 10 children diagnosed with TS, only 1 had a comorbid ADHD, using the DSM-III criteria and Conners scale. In this study, the rates of other neurodevelopmental disorders were also reported: 22% had comorbid ASD using the autism spectrum screening questionnaire (ASSQ) (5% Asperger, 17% PDD-NOS), 36% had comorbid dyslexia and 24% had a comorbid developmental coordination disorder. Another epidemiological study conducted in Sweden [20] on a population of 4479 children measured a 0.6% frequency of TS. In the TS group, the rate of comorbid ADHD, evaluated using the DSM-IV criteria and Conners scale, was 68% (60% combined subtype, 8% hyperactive-impulsive subtype); the rate of ASD was 20% (16% Asperger, 4% PDD-NOS); the rate of dyslexia was 16%; and the rate of developmental coordination disorder was 20%.

**Table 1:** Studies based on general population samples

Author (year)	N	Context	Age	Prevalence of TS (%)	Comorbidity	Scales	Nationality
Comings and Comings (1990) [16]	3034	School	NS (children)	0.46	ADHD 10/14 (70%)	NS	USA
Apter et al. (1993) [17]	2837	Israeli defense force recruitment	16–17	0.04	ADHD 8.3% OCD 41.7%	NS	Israel
Kadesjo and Gillberg (2000) [19]	435	School	Mean, 11	1.1	ADHD 1/11 (9%) Asperger 1/11 (9%) Dyslexia 2/11 (18%)	DSM III criteria Conners ASSQ	Sweden
Wang et al. (2003) [18]	2000	School	6–12	0.56	ADHD 36%	YSTSOBS	Taiwan
Khalifa and von Knorring (2006) [20]	4479	School	7–15	0.6	ADHD 68% (ADHD C 60%, ADHD HI 8%) PDD 20% (Asperger 16%, PDD-NOS 4%) OCD 16% Depression 20% Conduct disorder 8% Sleep disorder 28% DCC 20% LD 16% ID 16%	DSM IV criteria ASSQ CYBOCS Conners CBCL CDI	Sweden

ADHD, attention deficit hyperactivity disorder; ASSQ, autism spectrum screening questionnaire; CBCL, child behavior checklist; CDI, children’s depression inventory; CYBOCS, children’s Yale-brown obsessive compulsive scale; DCD, developmental coordination disorder; ID, intellectual disability; LD, learning disorder. NS, not specified; OCD, obsessive compulsive disorder; PDD-NOS, pervasive developmental disorder-not otherwise specified; YSTSOBS, Yale schedule for Tourette syndrome and other behavioral syndromes

### Clinical-based studies

#### Tourette syndrome and ADHD

ADHD is a neurodevelopmental disorder in which a persistent pattern of inattention and hyperactivity-impulsivity interferes with development and has a negative impact on social, academic or occupational functioning [1]. The diagnostic criteria and disease definition have changed over time and differ according to the system of classification used: in DSM-5, two dimensions are defined (hyperactivity and impulsivity versus inattention), and a diagnosis can be made if a minimum number of symptoms are scored in only one dimension specifying the subtype (hyperactive-impulsive subtype, combined subtype and predominantly inattentive subtype) [1, 21]. Worldwide prevalence of ADHD in children and adolescents using the DSM-5 broad definition is estimated to be between 5.29 and 7.1% [22]. The increased rates of prevalence of ADHD reported in the US over the past several years have led to the impression that ADHD is an “American disorder” and that it is much less prevalent elsewhere. The authors explained this difference primarily using the different methodological characteristics of the studies assessing prevalence rather than cultural or social factors [23]. Considering this variability in estimating the prevalence of ADHD, in our review we underline the nationality of the study and the methodology used to assess this comorbidity.

ADHD is the most common comorbid condition in patients with TS, as evidenced by the vast literature on the subject, with first association reports dating as early as 1973 [24]. 24 studies reported detailed data on ADHD in TS from clinical centres located worldwide (US, Australia, Japan, Iran, Brazil, England, Italy, Germany, Poland, and Denmark) (Table 2). The authors reported a 17–68% prevalence of ADHD in TS cohorts. This variability in comorbidity rate is not attributable to the geographic origin of the study, with US studies reporting similar ADHD comorbidities to studies conducted elsewhere. Of the studies selected, seven used specific rating scales to evaluate comorbid ADHD (e.g., Conners scales, an instrument used to assess ADHD and its most common comorbid problems). Other studies used screening questionnaires to assess behavioural and emotional problems (e.g., child behavior check list-CBCL) or semi-structured diagnostic interview or clinical evaluations based on the DSM criteria. Only 7 studies used a case–control methodology, and 2 [25, 26] differentiated among the three ADHD subtypes.

**Table 2:** Comorbidities in TS from studies based on clinical samples

Author (year)	N	Age	Methods used to evaluated comorbidities	Comorbidity prevalence rate	Main findings	Nationality
Comings and Comings (1987) [32]	246	Mean, 18.2	DSM III-based questionnaire	ADHD 48.8% ADD 11.8% Dyslexia 27%	TS patients have a significant risk for school problems and increased rate of dyslexia	USA
Chee et al. (1994) [54]	50	Mean, 20.8	Structured NS interview	ADHD 32% Depression 18% Anxiety 30%	Rate of prevalence of TS and comorbidities in an Australian TS cohort	Australia
Abwender et al. (1996) [55]	138	Children	NS	LD 22%	School difficulties are associated with comorbid ADHD	USA
Cardoso et al. (1996) [56]	32	Mean, 24	DSM IV criteria	ADHD NS 63% OCD 44% Sleep disorders 53% Depression 31% Impulse control deficit 28%	Rate of prevalence of TS and comorbidities in a Brazilian TS cohort	Brazil
Schuerholz et al. (1996) [57]	65	6–14	NS	LD 23%	LD is strongly correlated to the presence of ADHD	USA
Yeates et al. (1996) [34]	70	6–18	WRAT-R WCST HRB HRB WISC-R	Deficit in arithmetic 14/70 (20%) Deficit in reading (14%) General academic deficit (29%)	TS children classified in different learning disability subtypes reveal significant differences in neuropsychological functioning	USA
Wodrich et al. (1997) [58]	33	Children	DSM III criteria	ADHD NS 55% Depression 73% Conduct problems 18%	Prevalence and manifestations of comorbidities of TS patients in psychiatry practice are not identical to those reported in the literature	USA
Kano et al. (1998) [59]	64	Mean, 17.4	DSM III-R criteria	ADHD NS 17.2% OCD 62.5%	Rate of coprolalia in Japan is higher than the previously reported rate, and TS is often associated with developmental disorders	Japan
Freeman et al. (2000) [36]	3500	NS	DSM III/IV criteria	ADHD HI 7% ADHD C 51% ADHD PI 37% PDD 4.6% OCD 22.3% Mood disorder 16.9% Anxiety disorder 16.8% ID 3.4% Anger 27.6% Sleep problem 17.8%	ADHD is associated with an earlier diagnosis of TS and a higher rate of comorbidities (with the exception of anxiety disorders) One-third of TS + ADHD cases have LD, 26% have social skill deficits, and 39% have problems controlling anger	International database (author from Canada)
Teive et al. (2001) [33]	44 CTD (75% TS)	3–60	DSM IV criteria	ADHD 38.6% OCD 59.1% Affective disorders 11% Anxiety disorder 9% LD 14% Sleep disorder 9% Behavioural disorder 7%	Rate of comorbidities in a Brazilian clinical cohort	Brazil
Burd et al. (2005) [35]	5450	NS	DSM IV criteria	LD 22.7% ADHD NS 58%	In TS + LD, 80.2% patients also have ADHD and in the TS − LD group, 51.3% have ADHD; 31% of subjects with ADHD have also a diagnosis of LD	TIC international database (author from USA)
Saccomani et al. (2005) [60]	48	Mean, 11.2	DSM IV criteria	ADHD ns 43.8% OCD 54.2% Anxiety disorders 2.1% Sleep problems 27.1% Mood disorders 18.8%	Rate of comorbidities and clinical features of an Italian clinical cohort	Italy
Termine et al. (2006) [61]	17	Mean, 11.4	CBCL Conners SAFA K-SADS YGTSS	ADHD ns 11.8% ADHD ns + OCD 23.5% OCD 41.2%	TS patients have a high prevalence of ADHD and OCD compared with controls	Italy
Janik et al. (2007) [62]	126	Mean, 7.6	NS	ADHD ns 59%	Rate of comorbidities and clinical features of a Polish clinical cohort	Poland
Roessner et al. (2007) [63]	5060 (TIC database)	NS	DSM IV criteria	ADHD ns 61.2%	Comorbid ADHD is associated with high rates of externalising and internalising problems	International database (author from Germany)
Robertson et al. (2008) [64]	410	3–59	DSM IV criteria NHIS	ADHD 56% (230/410)	Factor analytic study. TS can be disaggregated into more homogeneous symptom components	USA
Ghanizadeh et al. (2009) [65]	35	Mean, 11.8	CBCL K-SADS YGTSS	ASD 2.9% ADHD 68.6%	Rate of comorbidities and clinical features of an Iranian clinical cohort	Iran
Gorman et al. (2010) [66]	65	Mean, 18	CBCL K-SADS CGAS CYBOCS Vineland YGTSS	ADHD 43% LD 27% OCD 25% Conduct disorder 15% Major depressive disorder 40%	Tic and ADHD severity are associated with a poorer psychosocial outcome	USA
Specht et al. (2011) [28]	126 (93.7% TS)	Mean, 11.7	ADIS-RLV CBCL CGI-S YGTSS CGAS	ADHD ns 26% Social phobia 21% Anxiety disorder 20% OCD 19%	In a sample of youth seeking treatment for a chronic tic disorder, ADHD is much lower than in clinically ascertained case series	USA
Lebowitz et al. (2012) [29]	158 CTD (143 TS)	6–14.5	Conners ASQ-P DISC IV CYBOCS MASC YGTSS CGAS	ADHD 38.6% OCD 53.8% ADHD + OCD 24.1%	TD with comorbid ADHD is associated with higher psychosocial stress and more externalising behaviours	USA
Rizzo et al. (2014) [67]	92	7–17	Conners DSM IV/V criteria CBCL MASC CDI YQLI-RV WISC-III	ADHD 22.2% ADHD + OCD 6.5% OCD 21.5%	TS + comorbidity patients have overrepresented affective and anxiety symptoms	Italy
Byler (2015) [30]	482 Two evaluation	Mean, 9.8	NS	ADHD 40% (first evaluation) + 21% (second evaluation) LD 5.4% Asperger 3% (first) + 2.1% (second) OCD 17% (first) + 14% (second evaluation) Survey: ADHD ns 41%, OCD 42%, LD 26.5%	More than 40% of TS patients continued to report ADHD or OCD as adults	USA
Hirschtritt et al. (2015) [9]	1374	Mean, 19.1	K-SADS DSM-IV structured interview	ADHD ns 54.3% OCD 66% Mood disorders 30% Disruptive behaviour 30% Anxiety 32%	ADHD began before tic onset and increased the presence of other comorbidities	USA Canada Great Britain Netherlands
Eapen et al. (2016) [12]	83	N = 43 < 18 N = 40 > 18	NHIS YGTSS HR-QoL	ADHD ns 21% LD 24% ASD 15% OCD 35% Anxiety disorder 45% Depressive disorders 33% Conduct disorder 4.8%	Presence of comorbidities and ADHD, in particular, has a greater impact on quality of life	Australia
Huisman-van Dijk et al. (2016) [11]	225	6–72	Conners AQ SCID-I CYBOCS YGTSS	ADHD 26% ASD 20% OCD 35.9%	Exploratory factor analyses (EFA) reveal a five-factor structure	Germany
Sambrani et al. (2016) [26]	1032 CTD (978 TS)	N = 529 < 18 N = 503 > 18	DSM IV criteria	ADHD 45% Hyperactivity 28.4% Inattention 39.4% OCD 10% Sleeping problems 27% Anxiety 31% Depression 23%	Comorbid ADHD reduces the patients’ ability for tic suppression	Germany

ADD, attention deficit disorder; ADHD, attention deficit/hyperactivity disorder; ADHD C, ADHD combined; ADHD HI, ADHD hyperactive; ADHD PI, ADHD predominantly inattentive; ADHD HADIS-RLV, anxiety disorders interview schedule for DSMIV: research and lifetime version for children and parents; ASD, autism spectrum disorder; ASQ, autism screening questionnaire; ASQ-P, Conners abbreviated symptom questionnaire-parent; ASSQ, autism spectrum screening questionnaire; AQ, autism-spectrum quotient; CBCL, child behavior checklist; CDI, children’s depression inventory; CGAS, children’s global assessment scale; CGI-S, clinical global impression-severity scale; CTD, chronic tic disorder; CYBOCS, children’s Yale-brown obsessive compulsive scale; DISC IV, diagnostic interview schedule for children; GTS-QOL, Gilles de la Tourette syndrome-quality of life scale; HRB, Halstead-retain neuropsychological test battery; ID, intellectual disability; K-SADS, Kiddie schedule for affective disorders and schizophrenia; LD, learning disorder; MASC, multidimensional anxiety scale for children; NHIS, national hospital interview schedule; NS, not specified; OCD, obsessive compulsive disorder; PDD, pervasive developmental disorder; PIC, personality inventory for children; SAFA, self administrated psychiatric scales for children and adolescents; SCID, structured clinical interview; STSS, Shapiro Tourette syndrome severity; TS, Tourette syndrome; WCST, Wisconsin card sorting test; WISC-R, Wechsler intelligence scale for children-revised; WRAT-R, wide range achievement test-revised; YGTSS, Yale global tic severity scale; YQLI-RV, youth quality of life-research; YSTSOBS, Yale schedule for Tourette’s syndrome and other behavioral syndromes

The presence of comorbid ADHD appears to be one of the most important determinants of quality of life and, in the presence of comorbid ADHD, the rates of other comorbidities (e.g., rage, symptoms of seasonal affective disorder, sleep disturbances and depressive symptoms) are significantly higher than in ‘TS only’ patients and contributes to poorer psychosocial outcome and educational problems [9, 27–29]. Comorbid ADHD has an important effect on prognosis in TS adult patients: Byler et al. [30] conducted a two-step analysis of a clinical cohort (using revision charts and then telephone surveys) finding that in adult life, more than 80% of TS patients reported persisting motor and vocal tics as mild or inexistent, but that more than 40% continued to report some type of comorbidity, with ADHD and OCD most commonly reported.

All these findings were confirmed in a large study conducted using the international “TIC” (Tourette Syndrome International Consortium) database containing 6805 TS patients from 22 countries [25]. The prevalence of ADHD in TS was stated to be 55% using the DSM-IV criteria. Regarding the preliminary data on 153 sequential cases, the authors could differentiate between ADHD subtypes, and they found the relative proportions of the three ADHD subtypes to be consistent with other studies on ADHD (7% fit the hyperactive-impulsive subtype, 51% fit the combined subtype and 37% fit the predominantly inattentive subtype). Comorbidity with ADHD was associated with earlier diagnosis and a higher rate of other comorbidities with the exception of anxiety disorder. Behavioural difficulties in TS with ADHD were found to be associated with the combined or hyperactive subtypes. Moreover, the authors found that comorbid ADHD was associated with a higher rate of developmental coordination disorder (14% in TS plus ADHD group versus 7% in TS without ADHD group).

#### Tourette syndrome and learning disorders

According to the DSM-5, LD are developmental disorders that begin by school age and impede the ability to learn or use specific academic skills (e.g., reading, writing, or arithmetic), which constitute the foundation for other academic learning [1]. The prevalence of LD in the general population is estimated to range between 5 and 9% [31]. School problems are frequently reported in TS children. Comings and Comings [32], summarising the different areas that impact on TS patients and cause school problems, individuated the most important as follows: motor tics that interfere in reading and writing; comorbidity with ADHD; deficits in socialization and rejection by peers and/or the teacher; medications that can cause cognitive blunting and contribute to learning problems; and the presence of specific LD.

The prevalence of LD in TS samples is only based on 10 studies (Table 2) including 4 that were specifically addressed to evaluate LD in TS patients; in the other papers, the focus was mainly on comorbidities in general. With the exception of 2 studies reporting a 5.4 and 14% prevalence of LD [30, 33], the other studies defined a more homogeneous prevalence (approximately 20–30%) of LD in TS patients (Table 2). Among the 9 studies selected, the diagnosis of LD was confirmed using the DSM criteria with only 2 studies utilising specific instruments for the assessment of LD.

Comings and Comings [32], evaluating a population of TS patients using a specific questionnaire for reading problems, found dyslexia in 26.8% of TS patients compared with 4.2% of the controls. Yeates et al. [34] examined the neuropsychological profiles of 70 TS children and classified them into four groups based on their pattern of performance on the wide range achievement test-revised (Table 2). Burd et al. [35], analysing the “TIC” database (5450 subjects), established a 22.7% prevalence of LD using the DSM criteria and compared TS subjects with comorbid learning disability and TS only subjects. The “TS plus LD” group showed an increased proportion of males, an earlier age of onset of TS, an earlier age at the time of first evaluation and diagnosis, a higher rate of perinatal problems, more severe tics and a higher rate of ADHD (80%).

#### Tourette syndrome and other comorbidities

Although this is not the focus of this review, we summarise the main results regarding the rates of other comorbidities rather than neurodevelopmental disorders obtained from the papers we selected. Obsessive compulsive disorder (OCD) together with ADHD is the most frequent comorbidity in TS patients with rates ranging from 10 to 60% [9, 26, 30]. In most studies, as demonstrated for ADHD, comorbidity with OCD represented one of the main determinants in terms of psychosocial and psychological outcomes in TS patients. Other comorbid conditions often reported in TS patients are depression (ranging from 11 to 73%), anxiety (2–45%), sleeping problems (9–53%) and externalising disorders or behaviours, such as conduct problems and rage attacks (5–30%). With the exception of anxiety, which is still debatable, these comorbidities appear to be highly associated with the presence of ADHD. Of the studies selected, only one [36] evaluated the presence of intellectual disability at a rate of 3.4%.

### Tourette syndrome and autism spectrum disorder

ASD, as classified by the DSM-5, is a neurodevelopmental disorder characterised by persistent deficits in social communication and social interaction across multiple contexts together with restricted and repetitive patterns of behaviour and interests or activities [1]. In a 2012 review, the global prevalence of ASD was estimated in a median of 62 cases per 10,000 people [37]. Tic disorders in ASD patients were first described in single case reports and in small case series [38–41]. Only few studies have analysed the prevalence of comorbid TS in large ASD clinical samples [42–44]. The reported TS rate in ASD population varies from 2.6 to 11% (Table 3). A transient association between TS and ASD has also been reported in a case series: Zappella described 12 young patients with early-onset TS comorbidity with reversible autistic behaviours. However, comorbidities between TS and ASD in most cases persist over time, and cases of ASD in TS samples have also been described. Clinical studies from TS samples highlight a prevalence of ASD in TS patients that can vary from 2.9 to 20% [11].

**Table 3:** Studies reporting ASD in TS samples and TS in ASD samples

Author (year)	Type of study	N	Age	Comorbidity rate	Scales	Country
TS in ASD samples						
Canitano and Vivanti (2007) [44]	Clinical cohort of ASD	105	Mean, 12	11% TS	DSM IV criteria YGTSS Vineland ABS	Italy
Baron-Cohen (1999) [43]	Clinical cohort of ASD	458	Mean, 11.1	6.2% TS	NHIS YGTSS DSM III-IV criteria	England
Kano et al. (1987) [42]	Clinical cohort of ASD	76	NS	2.6% TS	NS	Japan
ASD in TS samples						
Burd et al. (2009) [45]	Clinical cohort of TS	7288	NS	4.6% ASD	DSM IV criteria	Tic international database (author from US)
Ghanizadeh et al. (2009) [65]	Clinical cohort of TS	35	Mean, 11.8	2.9% ASD 68.6% ADHD	CBCL K-SADS YGTSS	Iran
Huisman-van Dijk et al. (2016) [11]	Clinical cohort of TS	225	6–72	26% ADHD 20% ASD 35.9% OCD	Conners AQ SCID-I Y-BOCS YGTSS	Germany

AQ, autism-spectrum quotient; ASD, autism spectrum disorder; CBCL, child behavior checklist; CYBOCS, children’s Yale-brown obsessive compulsive scale; K-SADS, Kiddie schedule for affective disorders and schizophrenia; NHIS, national hospital interview schedule; NS, not specified; OCD, obsessive compulsive disorder; SCID, structured clinical interview; Vineland ABS, Vineland adaptive behavior scales; Y-BOCS, Yale-brown obsessive–compulsive scale; YGTSS, Yale global tic severity scale

From the 6 studies found in the literature (Table 3), only one assessed the comorbidity between TS and ASD using a specific scale for autism (e.g., autism-spectrum quotient, AQ; childhood autism rating scale, CARS). Among the abovementioned clinical sample-based studies, the largest clinical sample of TS patients reported the most accurate characterisation of ASD comorbidity in our opinion [45]. Analysing 7288 patients from the Tourette Syndrome International Database Consortium Registry, the authors found that 334 (4.6%) TS individuals had comorbid ASD. In patients with TS and comorbid ASD, the rate of additional comorbidities increased considerably (98.8% TS + ASD patients had one or more comorbidities compared to 13.2% in the participants with TS only). A possible limitation of this work is represented by the fact that the diagnosis of ASD was confirmed using a structured reporting format based on the DSM criteria instead of an evaluation using specific instruments.

## Discussion

**Fig. 2:**
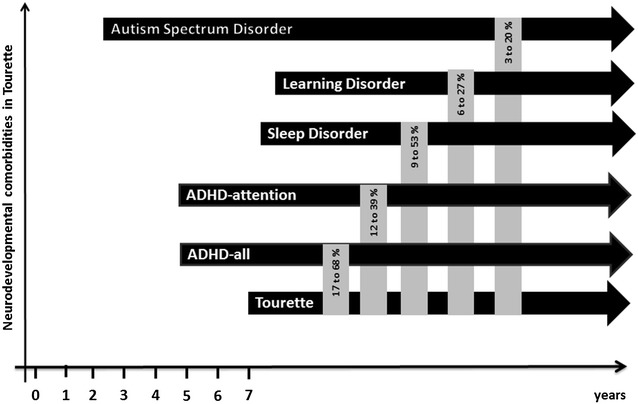
Co-occurrence of neurodevelopmental disorders in TS clinical samples according to age of onset and frequency

A peculiar feature of TS, which is now classified among the DSM-5 neurodevelopmental disorders, is represented by the frequent association with different comorbidities occurring in the majority of patients. In this paper, we reviewed the literature on comorbidity between TS and other DSM-5 neurodevelopmental disorders, focussing on ADHD, autism spectrum disorders and learning disabilities. Communication disorders and developmental coordination disorders were not included in this review because the scientific literature on this topic is still lacking. The available evidence suggests that neurodevelopmental comorbidities in TS are the rule rather than the exception. The high rate of neurodevelopmental comorbidities is found in general population studies as well as in clinical sample studies (Tables 2, 3). Among these neurodevelopmental comorbidities, ADHD is by far the most frequent. Considering the literature analysed, ADHD is one of the main determinants in terms of quality of life, psychosocial and psychological outcome. We found only few studies in which the abovementioned association distinguished the various ADHD subtypes. These studies demonstrated high rates of association with the combined and prevalent inattentive subtypes. Although we are aware that searches on TS and ASD as well as TS and LD produced few publications, they confirm an association between these disorders and support the idea of a continuum between simple TS without neurodevelopmental comorbidities and more complexes phenotypes (TS + ADHD + LD and TS + ASD). Other limitations of this review are the small number of studies or samples regarding several neurodevelopmental comorbidities (e.g., ASD) and the frequent lack of adequate diagnostic instruments for assessing patients and defining comorbidities. In particular, a further bias caused by the different quality of the studies evaluated, could lie in the absence of specific assessments performed by clinicians expert in the field, thus resulting in a possible overestimation of the rates of comorbidities, such LD and ASD [46]. Moreover, we are aware that the variability of the age of the populations investigated is a factor affecting the prevalence rate. However, the current results favour the inclusion of TS among neurodevelopmental disorders as a DSM-5 group of conditions. The inclusion criteria were based on prevalence, clinical characteristics and prognosis. Interestingly, TS not only co-occurs often with other neurodevelopmental disorders, but it is also likely linked through common genetic background and common risk factors, as evidenced by the recent literature [11, 13, 14, 47, 48]. Figure 2 provides a graphical representation of this spectrum of neurodevelopmental comorbidities based on the data obtained in TS clinical samples and according to frequency and age of onset. In this sense, it appears advisable to explore clusters of neurodevelopmental problems rather than screen them separately. This is consistent with the concept of “ESSENCE” (early symptomatic syndromes eliciting neurodevelopmental clinical examinations), a term coined by Gillberg to refer to children with major difficulties in one or more of the following fields: general development, communication and language, social inter-relatedness, motor coordination, attention, activity, behaviour, mood, and sleep [49].

From an empirical research perspective, additional studies that specifically address the comorbidity between TS and other neurodevelopmental disorders, use appropriate assessment instruments and design and do not neglect certain areas are warranted. First, studies evaluating the co-occurrence of all neurodevelopmental disorders as classified in DSM-5 (thus also including developmental coordination disorder, communication disorders and intellectual disabilities) in the same TS patients’ sample and in a developmental perspective are needed. Second, additional studies should address ADHD for a better characterization of comorbid ADHD into different subtypes. Similarly, subtypes of LD should be determined, using objective neuropsychological assessment of both attention and executive functions or specific learning investigations, such as literacy or calculation assessments. Third, from the literature analysed, it appears that most patients with ASD found in TS samples correspond to patients previously classified in DSM-IV as PDD-NOS or to patients affected by MCDD (multiple complex developmental disorder), a term proposed by Cohen et al. in 1986 [50] to refer to a group of children with atypical development. In this sense, more detailed studies focussing on comorbid ASD using developmental and dimensional perspectives are recommended.

In conclusion, a prompt diagnosis of comorbidities in TS patients and a characterisation of them in a more comprehensive approach are important not only to understand the aetiological basis of neurodevelopmental disorders but also, as clinical relevance, for a prompt definition of rehabilitative and therapeutic approaches. These include pharmacotherapy [51, 52] and, equally importantly, cognitive-behavioural therapy and social interventions [53].

## Abbreviations

ADHD: attention deficit/hyperactivity disorder
ADIS-RLV: anxiety disorders interview schedule for DSMIV: research and lifetime version for children and parents
ASD: autism spectrum disorder
ASQ: autism screening questionnaire
ASQ-P: Conners abbreviated symptom questionnaire-parent
AQ: autism-spectrum quotient
ASSQ: autism spectrum screening questionnaire
CBCL: child behavior checklist
CDI: children’s depression inventory
CGAS: children’s global assessment scale
CGI-S: clinical global impression-severity scale
CTD: chronic tic disorder
CYBOCS: children’s Yale-brown obsessive compulsive scale
DCD: developmental coordination disorder
DISC IV: diagnostic interview schedule for children
GTS-QOL: Gilles de la Tourette syndrome-quality of life scale
HRB: Halstead-retain neuropsychological test battery
ID: intellectual disability
LD: learning disorder
K-SADS: Kiddie schedule for affective disorders and schizophrenia
MASC: multidimensional anxiety scale for children
NHIS: national hospital interview schedule
NS: not specified
OCD: obsessive compulsive disorder
PDD-NOS: pervasive developmental disorder-not otherwise specified
PIC: personality inventory for children
SAFA: self administrated psychiatric scales for children and adolescents
SCID: structured clinical interview
STSS: Shapiro Tourette syndrome severity
TS: Tourette syndrome
Vineland ABS: Vineland adaptive behavior scales
WCST: Wisconsin card sorting test
WISC-R: Wechsler intelligence scale for children-revised
WRAT-R: wide range achievement test-revised
Y-BOCS: Yale-brown obsessive–compulsive scale
YGTSS: Yale global tic severity scale
YQLI-RV: youth quality of life-research
YSTSOBS: Yale schedule for Tourette’s syndrome and other behavioral syndromes
